# Application of the screening test principles to screening for neonatal hypoglycemia

**DOI:** 10.3389/fped.2022.1048897

**Published:** 2022-12-07

**Authors:** J. M. Alsweiler, N. Heather, D. L. Harris, C. J. D. McKinlay

**Affiliations:** ^1^ Department of Paediatrics: Child and Youth Health, University of Auckland, Auckland, New Zealand; ^2^ Newborn Metabolic Screening Programme, LabPlus, Te Whatu Ora Te Toka Tumai Auckland, Auckland, New Zealand; ^3^ Liggins Institute, University of Auckland, Auckland, New Zealand; ^4^ School of Nursing, Midwifery and Health Practice, Faculty of Health, Victoria University of Wellington, Wellington, New Zealand

**Keywords:** neonatal, hypoglycemia, screening, neurodevelopment, neuroglycopenia

## Abstract

Severe and prolonged neonatal hypoglycemia can cause brain injury, while the long-term consequences of mild or transitional hypoglycemia are uncertain. As neonatal hypoglycemia is often asymptomatic it is routine practice to screen infants considered at risk, including infants of mothers with diabetes and those born preterm, small or large, with serial blood tests over the first 12–24 h after birth. However, to prevent brain injury, the gold standard would be to determine if an infant has neuroglycopenia, for which currently there is not a diagnostic test. Therefore, screening of infants at risk for neonatal hypoglycemia with blood glucose monitoring does not meet several screening test principles. Specifically, the long-term neurodevelopmental outcomes of transient neonatal hypoglycemia are not well understood and there is no direct evidence from randomized controlled trials that treatment of hypoglycemia improves long-term neurodevelopmental outcomes. There have been no studies that have compared the long-term neurodevelopmental outcomes of at-risk infants screened for neonatal hypoglycemia and those not screened. However, screening infants at risk of hypoglycemia and treating those with hypoglycaemic episodes to maintain the blood glucose concentrations ≥2.6 mmol/L appears to preserve cognitive function compared to those without episodes. This narrative review explores the evidence for screening for neonatal hypoglycemia, the effectiveness of blood glucose screening as a screening test and recommend future research areas to improve screening for neonatal hypoglycemia. Screening babies at-risk of neonatal hypoglycemia continues to be necessary, but as over a quarter of all infants may be screened for neonatal hypoglycemia, further research is urgently needed to determine the optimal method of screening and which infants would benefit from screening and treatment.

## Introduction

Neonatal hypoglycemia is common with 50% of at-risk infants, 15% of all newborns, having one or more low blood glucose concentrations in the first 48 h after birth (1). Severe or prolonged hypoglycemia, while rare, can cause severe brain injury with lifelong disability (2). Transitional hypoglycemia, defined as low blood glucose concentrations in otherwise well late preterm and term neonates, in the absence of metabolic, endocrine or genetic disorders (3), is much more common, and not confined to infants with risk factors. Although the long-term effects are less well understood (4), transitional hypoglycemia is associated with adverse effects on neurodevelopment (5). Neonatal hypoglycemia is commonly asymptomatic, and it is standard practice to screen infants considered at increased risk, including infants of mothers with diabetes (IDM) and those born preterm, large and small for gestational age (LGA, SGA), with serial blood tests in the first 12–24 h after birth (6). However, what began 50 years ago as a pragmatic measure in a small proportion of IDM or growth restricted infants, has expanded through changes to the diagnostic thresholds, expansion of the risk criteria and a rapid increase in the incidence of diabetes in pregnancy to become a routine targeted screening test in more than a quarter of newborn infants, without having been evaluated or formally implemented as a screening programme.

## Population screening

Medical screening is the systematic application of a test or inquiry, to identify individuals at sufficient risk of a specific disorder to benefit from further investigation or direct preventive action, among those who have not sought medical attention on account of symptoms of that disorder (7). Targeted or selective screening is the screening of high-risk groups in the population, which may still be at large scale and can be considered as a form of population screening.

All screening is harmful to some degree, and costly, so before a screening programme is introduced it should meet the principles of a screening programme to ensure that the screening will be more beneficial than harmful (8–10). The original principles outlined by Wilson and Jungner in 1968 (8) have been updated and modified (10–12). The key principles of a screening programme include that the condition should be important, and its natural history well understood; there should be a simple safe and validated test, with an agreed, well defined, cut-off threshold which is acceptable to the target population; the intervention should be effective at improving outcomes when given in the pre-symptomatic phase; and the screening programme should have a clear objective, with data from randomized controlled trials demonstrating its effectiveness at improving outcomes, be equitable and well-resourced with good quality assurance measures.

In the current era there are rigorous steps to ensure a new screening programme meets these criteria before a screening programme is introduced. National or state committees rigorously review the evidence before recommending the introduction of a new screening programme, including screening tests for newborn infants (13). Newborn screening includes population screening not only for various metabolic, immune and endocrine conditions through a routine blood test after birth, but also screening for congenital cyanotic heart disease using pulse oximetry (14); hearing impairment using auditory stimulation; and screening for cataracts and developmental dysplasia of the hip by newborn examination. Some of the conditions screened for in the newborn screening programme, e.g., medium-chain acyl-CoA dehydrogenase deficiency, can cause neonatal hypoglycemia. However, targeted screening of newborn infants at high-risk for neonatal hypoglycemia, including IDM, preterm, SGA and LGA infants, who make up around 30% of all newborn infants (15), is not officially included in national newborn screening programmes.

## Screening for neonatal hypoglycemia

### History

Symptomatic neonatal hypoglycemia was first associated with poor neonatal outcomes in 1959 (16). In 1965, Cornblath and Reisner described that glucose has been measured in the blood of newborns since 1911, but “there is still disagreement over which levels of blood sugar are normal in the neonate and which are hypoglycaemic or hyperglycaemic” (17). Cornblath and Reisner also noted that low blood glucose concentrations could be observed in IDM and premature infants without obvious symptoms, but that “whether or not low levels of glucose without clinical manifestations produced brain damage remained to be elucidated” (17). While neuroglycopenia causes harm, it still remains to be fully elucidated if asymptomatic neonatal hypoglycemia in at-risk infants is a reliable marker of neuroglycopenia sufficient to cause brain damage. However, evidence with a low grade of certainty, in at-risk asymptomatic infants tested and treated for hypoglycemia, shows an association between hypoglycemia and impaired neurodevelopment (4, 5).

Concern that asymptomatic hypoglycemia may lead to neurodevelopmental sequalae led to the introduction of widespread screening of asymptomatic at-risk (IDM, SGA and asphyxiated) infants for neonatal hypoglycemia in the 1970s (18). Unlike the recommendations for the introduction of screening tests in the modern era (9), a standardised process involving the generation of data from randomized controlled trials on the efficacy and cost-effectiveness of blood glucose concentration screening to prevent brain damage was not performed. Instead, screening for neonatal hypoglycemia was facilitated by the availability of point of care testing which led to the screening of an ill-characterised clinical entity, with little evidence that the infants involved benefitted from screening (18).

### Diagnostic thresholds and at-risk groups

In the 1950s, the initial thresholds below which low blood glucose concentrations would not be tolerated were less than 20 mg/100 ml (1.1 mmol/L) in growth restricted and 30 mg/100 ml (1.7 mmol/L) in well grown infants. There was wide variation in the definition of hypoglycemia in term infants among both textbooks and paediatricians in the 1980s, ranging from blood glucose concentrations of <1.0 to <4.0 mmol/L (19). In 1988, two studies defining neonatal hypoglycemia were published, one a secondary analysis of a randomized controlled trial of feeding in preterm infants (20), and the other a small observational study in 17 children, five of whom were neonates (21). Both studies found that a blood glucose concentration below 2.6 mmol/L was associated with worse neurodevelopmental outcomes. This led to a blood glucose concentration of 2.6 mmol/L being widely, although not uniformly, adopted as the blood glucose concentration threshold at which to define hypoglycemia (22). Subsequently, the Pediatric Endocrine Society published guidelines recommending that the threshold be 2.8 mmol/L in the first 48 h after birth, rising to 3.3 mmol/L thereafter (23).

The initial screening selected mainly IDM and SGA infants for blood glucose testing, with a frequency of low blood glucose concentrations of 4.4/1,000 live births in an era when diabetes in pregnancy was rare (24, 25). The criteria expanded over time to include LGA and preterm infants. In addition, some guidelines recommended testing infants of women with obesity and those exposed to maternal beta blocker or antenatal corticosteroid therapy. However, few guidelines recommend testing infants born to pregnant people with pre-eclampsia, despite the original paper from Cornblath in 1959 describing pre-eclampsia as a risk for hypoglycemia (16).

Data on the normal blood glucose concentrations of term newborns was originally published in the 1965, showing an initial dip after birth, with a lower mean blood glucose concentration for approximately the first 2 to 3 days (17). Subsequently, it was reported that 38% of uncomplicated term infants in Nepal had a blood glucose concentration of <2.6 mmol/L in first 50 h. In this paper, hypoglycemia was discussed as being a common problem in Nepal, rather than considering, that at 38% of normal births, this was potentially within the normal range (26). A recent study of uncomplicated term infants, appropriate weight for gestational age and born to non-obese mothers without diabetes confirmed that 39% of infants have at least one blood glucose concentration less than 2.6 mmol/L in the first 5 days after birth (27). This would mean that 40% of normal infants would be considered in need of treatment on the first day after birth according to recommendations by the Pediatric Endocrine Society (23).

## Does neonatal hypoglycemia meet the principles for a screening programme?

Screening for neonatal hypoglycemia does not meet the majority of the original or updated principles for a screening test (Table 1).

**Table 1:** Screening principles criteria applied to screening for neonatal hypoglycemia.

	Screening criteria			Neonatal hypoglycemia	
	Wilson and Jungner 1968 (8)	UK National Screening Committee: Targeted screening 2022 (10)	Andermann 2008 (12)	Comments	Meets the criteria
**The Condition**	1. The condition should be an important health problem.	1. The health impact of the condition and its course should be understood, with evidence that serious disease can be identified or predicted by an agreed level of identifiable risk or marker.	The screening programme should respond to a recognised need.	Severe neonatal hypoglycemia is rare but causes brain injury. Transitional hypoglycemia is common and associated with developmental delay.	Yes
	7. The natural history of the condition, including development from latent to declared disease, should be adequately understood.			The natural history of the condition i.e. the risk of brain injury from mild/transitional neonatal hypoglycemia, is poorly understood.	No
	4. There should be a recognizable latent or early symptomatic phase.			Even one low blood glucose concentration is associated with poor academic outcomes.	Maybe
**The Test**	5. There should be a suitable test or examination.	2. There should be evidence from appropriately designed studies and models on cost effectiveness for the: screening test – this should be a simple test that has evidence of suitable accuracy and technical performance derived from studies in the population in which the test is being used.		Blood glucose concentration testing is simple, although painful. Evidence that blood glucose testing with an enzyme method is cost-effective.	Yes
		3. There should be a diagnostic investigation available for individuals with a positive test screening result, with evidence that subsequent tests can distinguish those who would benefit from interventions from those who would not.		There is not a gold standard test for neuroglycopaenia for infants with a positive test screening result to determine which infants would benefit from intervention.	No
	6. The test should be acceptable to the population.			No data on acceptability of the test to parents or the infants (as adults).	No
**The Intervention**	2. There should be an accepted treatment for patients with recognised disease.	2. There should be evidence from appropriately designed studies and models on cost effectiveness for the: intervention – there should be better outcomes from early intervention/those at a pre-symptomatic stage, for the screened individual, compared with usual care.		Treatment options include oral dextrose gel, breast milk substitute, intravenous dextrose, diazoxide, glucagon. There is no direct evidence that any of these interventions in the pre-symptomatic phase improve neurodevelopment.	No
	8. There should be an agreed policy on whom to treat as patients.	5. There should be a robust and inclusive evidence-based selection criteria for identifying those eligible for targeted screening.	There should be a defined target population.	Target population varies from country to country, but all guidelines recommend screening IDM, preterm and SGA babies. Screening for LGA babies contentious.	Yes
**The Screening Programme**			The objectives of the screening programme should be defined at the outset.	No objectives defined.	No
			There should be scientific evidence of screening programme effectiveness.	No direct evidence of screening effectiveness from randomized controlled trials.	No
		4. The overall benefits from the screening programme should outweigh the harms, for example, from overdiagnosis, overtreatment, false positives, false reassurance and uncertain findings.	The overall benefits of screening should outweigh the harm.	The degree of benefit of neonatal hypoglycemia screening is uncertain and there is a possibility of harm.	No
	9. The cost of case-finding (including diagnosis and treatment of patients diagnosed) should be economically balanced in relation to possible expenditure on medical care as a whole.			No data available of the cost effectiveness of screening for neonatal hypoglycemia.	No
			The programme should promote equity and access to screening for the entire target population.	Indigenous and minority groups likely to be at higher risk due to higher rates of diabetes in pregnancy and SGA babies. No data on equity and access to screening.	No
**Implementation criteria**		7. The UK NSC should carry out a feasibility assessment (informed by NHS practice) that indicates the screening programme would be achievable; with evidence for monitoring and quality assuring the programme, adequate staffing and facilities being available to meet the requirements of programme delivery.	There should be quality assurance, with mechanisms to minimize potential risks of screening.	Clinical management of the condition not optimised as the definition of hypoglycemia and the glycaemic targets for treatment not established. Several *ad hoc* audits have been published, but no agreed set of quality assurance standards.	No
** **	10. Case-finding should be a continuing process and not a “once and for all” project.				Yes
			Programme evaluation should be planned from the outset.	No planned programme evaluation.	No
	3. Facilities for diagnosis and treatment should be available.		The programme should integrate education, testing, clinical services and programme management.	Neonatal hypoglycemia not included in newborn screening programmes.	No
		6. There should be evidence that the screening programme is acceptable to the public and health professionals, with appropriately balanced information available to those invited to attend screening.	The programme should ensure informed choice, confidentiality and respect for autonomy.	Some sites/countries will have pamphlets on neonatal hypoglycemia available for parents. Limited evidence of informed choice.	Maybe

IDM; infant of mother with diabetes, LGA; large for gestational age, NHS; National Health Service, SGA; small for gestational age, UK NSC; United Kingdom National Screening Committee.

### The condition

Neonatal hypoglycemia is an important health problem (2, 4). Neonatal hypoglycaemia is not a disease in its own right, but a symptom of multiple diseases. Most babies perceived to be at-risk of neonatal hypoglycaemia e.g infants of diabetic mothers, have transitional hypoglycemia due to prolonged postnatal adaptation. In addition, infants with hypoglycemia are all treated to increase the blood glucose concentration, using the same treatments, with the same goal, to prevent brain damage. The natural history of transitional hypoglycemia is reasonably well understood, with resolution and a slightly delayed inflexion point in metabolic transition. However, it is unclear under what conditions there is net clinical benefit from interventions aimed at increasing the blood glucose concentration. At-risk infants with neonatal hypoglycemia who were tested and treated to maintain their blood glucose concentration >2.5 mmol/L had similar risks for neurodevelopmental impairment to at-risk infants who did not develop neonatal hypoglycemia (28–30). However, there are no data on the natural history of infants who had blinded screening for neonatal hypoglycemia and were not diagnosed or treated. Infants in the CHYLD study had blinded CGM measurements of their interstitial glucose concentrations but were also tested and treated for hypoglycemia with intermittent blood glucose concentration monitoring. There was an increased risk of abnormal neurodevelopment in infants with undiagnosed low interstitial glucose concentrations at 4.5 years after birth (29), but not at 2 years after birth or in mid childhood (28, 30).

It is an important principle of screening that there is a recognizable latent or early symptomatic phase. Even one or two low blood glucose concentrations has been associated with neurocognitive impairment (31) and worse academic performance (32), suggesting that there may not be a recognizable latent phase in which treatment of neonatal hypoglycemia can prevent neurodevelopmental impairment. However, screening may be worthwhile even if the initial latent phase is missed if further harm can be prevented.

### The test

The test for neonatal hypoglycemia, a capillary blood glucose measurement, analysed by an enzymatic (glucose oxidase or hexokinase) method of analysis is relatively easy to obtain, although painful for the infant. However, there is no gold standard test for neuroglycopenia, a state of low glycolysis in neurons leading to excitotoxicity and reactive oxidative species and eventual neuronal death (33). Therefore, blood glucose concentrations cannot be validated as an effective screening test for neuroglycopenia. Moreover, while the distribution of test values in at-risk infants (IDM, preterm, SGA, LGA) is known (1) the threshold at which neonatal hypoglycemia is defined remains controversial. The accepted threshold ranges from <25 mg/100 ml (1.4 mmol/L) in the first 4 h (6) to <50 mg/100 ml (2.8 mmol/L) in the first 48 h (23). The uncertainty in international guidelines reflects the lack of evidence guiding the threshold at which neonatal hypoglycemia should be defined, as the frequently utilised threshold of 2.6 mmol/L is without scientific justification (6). However, there is reasonable indirect evidence that a blood glucose concentration threshold of 2.6 mmol/L is an adequate operational threshold provided it is used within a proactive framework of close blood glucose monitoring and protocol-based management (28).

### The intervention

There are several commonly used interventions to treat neonatal hypoglycemia. However, only a few of the interventions have been shown to be effective at treating neonatal hypoglycemia in randomized controlled trials, including oral dextrose gel (34, 35) and diazoxide (36). The effectiveness of other interventions, including breastmilk substitute, intravenous dextrose, and glucagon (37) has not been determined in randomized controlled trials. However, in a randomized controlled trial of different glycemic treatment targets, infants randomized to maintain a blood glucose concentration of ≥2.6 mmol/L using supplementary oral feeding, tube feeding or intravenous glucose administration, had higher blood glucose concentrations than infants randomized to maintain a blood glucose concentration of ≥2.0 mmol/L (38), confirming that these interventions increase blood glucose concentrations in hypoglycemic newborns. However, while there is evidence that treatment with oral dextrose gel reduces short-term harm (34), currently there is no direct evidence that any intervention for neonatal hypoglycemia improves long term neurodevelopmental outcomes, although treatment with dextrose gel reduces short-term harm (neonatal intensive care unit admission, breastmilk substitute use) without worsening developmental outcomes (39, 40). Of concern, there is some evidence that rapidly increasing the blood glucose concentration after neonatal hypoglycemia is associated with a higher incidence of neurosensory impairment (30, 41).

In addition to uncertainty on the threshold at which neonatal hypoglycemia is defined, there is also uncertainty about the glycemic target that should be maintained with treatment. While most guidelines recommend maintaining the blood glucose concentrations at 2.6 mmol/L, a lower target of 2.0 mmol/L is non-inferior based on neurodevelopmental assessment at 18 months corrected age (38).

Currently, testing for neonatal hypoglycemia is a targeted intervention, with only infants considered at risk for hypoglycemia offered screening. International guidelines are consistent in recommending that IDM, SGA and preterm infants are at increased risk for neonatal hypoglycemia, although the thresholds to define SGA and preterm vary between guidelines (42, 43). IDM and SGA are at increased risk of hyperinsulinaemic hypoglycemia, which not only reduces the blood glucose concentration, but may also reduce alternative cerebral fuels, with ketones largely absent in infants with hypoglycemia in the first 48 h after birth (44).

Whether LGA infants are at increased risk for neonatal hypoglycemia is more contentious, with only half of international/state guidelines considering them at increased risk such as to recommend testing (15). There is no evidence that otherwise healthy LGA infants are at increased risk of neurodevelopmental impairment due to neonatal hypoglycemia (45, 46). A review of cases of neonatal hypoglycemia resulting in brain damage which resulted in litigation, found that all the infants were either IDM or SGA, with none of the infants LGA (47).

### The screening programme

The principles for screening programmes assume that the programme is being applied for and is not yet in place and recommend that the objectives of a screening programme should be defined at the outset. However, screening for neonatal hypoglycemia “crept in through the back door”, and despite more than a quarter of all newborns being screened for neonatal hypoglycemia (15), it is not recognised as an official screening programme. Therefore, objectives are often vague or assumed to be the prevention of brain injury. There is also no evidence from randomized controlled trials that screening for neonatal hypoglycemia is effective at reducing mortality or morbidity and no evidence that the screening pathway, which includes multiple painful blood tests, is acceptable to health professionals or the public.

Raffle and Gray have been quoted as saying “All screening programmes do harm. Some do good as well and, of these, some do more good than harm at reasonable cost” (48). Therefore, a key principle of screening programmes is that the benefit gained from the screening programme should outweigh any harms. There is benefit from screening to identify at-risk infants for neonatal hypoglycemia. Severe or prolonged hypoglycemia causes brain injury (2, 47), and even mild or transitional hypoglycemia is associated with neurodevelopmental impairment although the evidence is of low certainty (4, 31). However, the degree of benefit is uncertain, and it is not known how many at-risk infants need to be screened to prevent one case of neurodevelopmental impairment.

As there have been no randomized trials in at-risk infants of testing compared to not testing for neonatal hypoglycemia, there are neither data on the benefits nor the harm of this approach, but there are likely to be harms in unnecessary testing of infants incorrectly identified as at increased risk for hypoglycemia. In addition to multiple painful blood tests, there are also concerns that introducing potentially unnecessary breastmilk substitute may reduce breastfeeding rates (49), contributing to infants at risk of hypoglycemia, such as IDM and preterm infants, being less likely to be exclusively breastfed on discharge from hospital (50, 51). In addition, infants may be potentially unnecessarily separated from their mothers.

Newborn screening programmes have previously been conflated by positive tests for a severe disease for those with a mild or benign variant which causes no long-term effects, with ill effects from the intervention as a result. For example, the first newborn screening programme was for phenylketonuria, where high concentrations of phenylalanine on a dried blood spot were predictive of the condition which could lead to severe intellectual disability if not recognised early and treated with a restricted diet. However, initially it was not recognised that people who are heterozygotes for the mutation have higher than normal concentrations of phenylalanine, which does not cause long term effects, but a restricted diet in these patients could be harmful (52). It is concerning that many infants that are screened for neonatal hypoglycemia, will have one or several low blood glucose concentrations, and as a result receive breastmilk substitute and/or intravenous dextrose, potentially be separated from their mother, resulting in failure to achieve full breastfeeding, with potentially long-term effects on health (53). However, they may have had transitional hypoglycemia that was self-resolving, with blood glucose concentrations less than 2.6 mmol/L common in healthy infants (27), with no effects on their long-term development, i.e., they have been harmed because they were tested for neonatal hypoglycemia.

The potential costs of neonatal hypoglycemia, including postnatal hospital costs and the costs of neurodevelopmental impairment are expensive (54). It is cheaper to use an accurate enzymatic point of care device than a less accurate non-enzymatic device which needs to have low results confirmed at the laboratory (55). Buccal dextrose gel as a treatment for neonatal hypoglycemia reduces the cost for management (56). However, the cost effectiveness of screening for neonatal hypoglycemia has not been established.

Screening is more likely to occur in higher socioeconomic groups with a lower risk of severe disease (9); therefore, it is important that screening programmes are designed to be equitable. There are few data available on whether neonatal hypoglycemia screening is equitable, although in one study there was no difference in adherence to neonatal hypoglycemia screening guidelines by ethnicity in a multi-ethnic population, although adherence was low overall (57). Nevertheless, indigenous and minority groups are likely to be at higher risk of neonatal hypoglycemia due to higher rates of risk factors, including diabetes in pregnancy (58) and preterm birth (59).

### Implementation

As with any medical intervention, a screening programme will only be as successful as its implementation. A screening programme should be feasible, with a quality assurance programme, adequate staffing and facilities being available to meet the requirements of programme delivery and should integrate education, testing, clinical services and programme management.

There are currently no agreed set of quality assurance standards for a neonatal hypoglycemia screening programme, nor a plan for monitoring or evaluating the programme. Several audits have shown low adherence to neonatal hypoglycemia guideline recommendations (57, 60). However, it is also important to consider the accuracy of blood glucose analysers, timeliness of results and, appropriate follow-up algorithms including actions after a low blood glucose measurement. Without a gold standard test it is not possible to define true and false screen-positives to calculate standard screening metrics including sensitivity, specificity and positive predictive value.

It is unknown if parents are given the opportunity to make an informed choice regarding neonatal hypoglycemia screening for their baby. While some risk factors are known during pregnancy, such as maternal diabetes, other risk factors, such as preterm birth or SGA, may only be recognised at the time of birth, giving parents little time to make an informed decision about testing for hypoglycemia, which is commonly recommended to begin in the first 1 to 2 h after birth.

## Future research areas to improve neonatal hypoglycemia screening

Screening at-risk infants for neonatal hypoglycemia is standard practice, and it would now be difficult, but not impossible, to conduct a randomized controlled trial of screening compared to no screening in infants at risk of neonatal hypoglycemia. Such a trial could be justified on the grounds of the high cost of screening, that screening may be causing harm, and that the current screening approach doesn't prevent severe hypoglycemia. There is a lack of consensus on whether LGA infants whose mothers do not have diabetes in pregnancy benefit from testing for neonatal hypoglycemia. Therefore, in contrast to other at-risk infants, it would be feasible to conduct a randomized controlled trial to assess whether screening and treating LGA infants for neonatal hypoglycemia improves their longer-term neurodevelopment.

It would also be difficult to conduct accurate retrospective studies on screening for neonatal hypoglycemia and neurodevelopmental outcome, as there would be significant confounders between babies who were tested for hypoglycaemia and those who were not tested. More research is also needed to determine the views of parents on screening their infants for neonatal hypoglycemia.

## Conclusions

Screening for neonatal hypoglycemia does not meet the principles for a screening test, due to inadequate data on the natural history of transitional hypoglycemia; lack of an agreed, evidence-based definition of damaging hypoglycemia; lack of high quality data on interventions that improve long-term outcomes, and an equitable and quality assured screening programme. However, as at-risk babies are at risk of significant brain injury, testing of babies at increased risk of neonatal hypoglycemia continues to be necessary. Further research is needed to determine which infants benefit from screening for neonatal hypoglycemia.
